# Using a Data-Driven Approach to Estimate Second-Language Proficiency From Brain Activation: A Functional Near-Infrared Spectroscopy Study

**DOI:** 10.3389/fnins.2020.00694

**Published:** 2020-07-10

**Authors:** Miaomei Lei, Toshinori Miyoshi, Ippeita Dan, Hiroki Sato

**Affiliations:** ^1^Research & Development Group, Hitachi, Ltd., Tokyo, Japan; ^2^Research and Development Initiatives, Applied Cognitive Neuroscience Laboratory, Chuo University, Tokyo, Japan; ^3^Department of Bioscience and Engineering, College of Systems Engineering and Science, Shibaura Institute of Technology, Saitama, Japan

**Keywords:** machine learning, language proficiency, brain activation, feature selection, native Japanese speakers, native English speakers

## Abstract

While non-invasive brain imaging has made substantial contributions to advance human brain science, estimation of individual state is becoming important to realize its applications in society. Brain activations were used to classify second-language proficiencies. Participants in functional near-infrared spectroscopy (fNIRS) experiment were 20/20 native Japanese speakers with high/low English abilities and 19/19 native English speakers with high/low Japanese abilities. Their cortical activities were measured by functional near-infrared spectroscopy while they were conducting Japanese/English listening comprehension tests. The data-driven method achieved classification accuracy of 77.5% in the case of Japanese speakers and 81.9% in the case of English speakers. The informative features predominantly originated from regions associated with language function. These results bring an insight of fNIRS neuroscience and its applications in society.

## Introduction

Language, which differentiates human beings from other living species, plays an important role in our daily lives. The neural basis of language has been investigated with various techniques for functional neuroimaging (Price, 2012; Quaresima et al., 2012). Functional near-infrared spectroscopy (fNIRS) is an optical neuroimaging technique that measures brain activity by monitoring the hemodynamic changes in cerebral cortex response of brain activation. Its main advantages are relatively low cost, portability, safety, low acoustic noise (compared to functional magnetic resonance imaging), and easiness to operate (Scholkmann et al., 2014; Hong and Yaqub, 2019). In the context of fNIRS community, hemodynamic changes (which represent brain activation) have been used as a useful indicator to demonstrate speech perception in infants (Pena et al., 2003; Bortfeld et al., 2009; Sato et al., 2012) and language comprehension in adults (Sato et al., 1999; Schecklmann et al., 2008; Lei et al., 2018). Since conventional analysis of fNIRS data has focused on human brain activity at the group level, these studies have traditionally drawn a population-level conclusion about general patterns across a large number of participants. Knowledge from these studies has important implications for advancing our understanding of how the human brain processes language. To further translate this knowledge into practical applications in society, individual estimation or classification of language ability (e.g., speech-comprehension level and second-language proficiency) on the basis of neuroimaging data across participants is a topic of interest.

Research interest in estimating the state of an individual by applying machine learning using fNIRS data has been increasing. fNIRS data with corresponding labels/classes are used to train a machine-learning classifier/model. The trained classifier is applied to the unknown data to estimate the labels. For example, in previous studies, mental arithmetic and music imagery (Power et al., 2010) motor imagery (Naseer and Hong, 2013), and subjective preference (Luu and Chau, 2008) were estimated. These studies showed the feasibility of establishing a predictive machine-learning model based on the state of individuals and their underlying brain activity. However, they focused on categorical discrimination to estimate the state of a participant using data of the participant. It is still a challenge to estimate the state of individuals on the basis of neuroimaging data of others, namely, estimation of state across participants. Clinical studies focusing on classification of diseases or disorders have made progresses in such estimation across participants (Hosseini et al., 2018; Sutoko et al., 2019) however, estimation of, for example, language ability, which may subtly differ across groups, remains unstudied.

The major difficulty concerning estimation across participants based on neuroimaging data is the relatively small data sample with individual differences. Individual differences refer to the variations across participants even though they have the same label in the same population group, for example, patients who have the same disease. It has been recognized that the brain structure and its corresponding function show high individual variability even among a healthy population group (Raz et al., 2005; Qin et al., 2014; Finn et al., 2015). From the viewpoint of machine learning, if input patterns have high individual differences, data in the feature space will be almost impossible to separate according to the label. It is thus difficult to construct a machine-learning classifier and model for the estimation, that is, separating feature vectors based on the information of the label. On the other hand, estimation with a small number of data samples is also a challenge; that is, the data dimensionality is usually much higher than the number of samples available for classifier training (Fan et al., 2007; An et al., 2017). This typical problem is known in machine-learning literature as the “curse of dimensionality” (Bellman, 2015). It may make the model unstable or cause the problem of overfitting (Guyon and Elisseeff, 2003), which is the condition that model fits accurately to the training data (including inherent noise) but fits poorly to unknown test data. In addition, in a practical situation, increasing the number of data samples is not always possible, for example, when the number of patients with a particular disease is limited. To solve this unbalance between number of features and sample size with the aim of increasing classification accuracy, various methods of feature selection (to extract a subset of most-informative features) have been proposed (Saeys et al., 2007; Pereira et al., 2009; Hu et al., 2013; Mwangi et al., 2014; Hong et al., 2018). Common methods of feature selection include using *t*-test (De Martino et al., 2008) ANOVA (analysis of variance) (Akama et al., 2014; Lei et al., 2014) Pearson correlation coefficient (Fan et al., 2007) and prior knowledge (Chu et al., 2012). Recently, sparse-feature selection has become one of the choices for data-driven feature selection (Tibshirani, 1996). Sparse techniques combine both machine learning and feature-reduction steps by enlisting a L1-norm regularization, resulting in a reduced subset of relevant features (Zou and Hastie, 2005).

In our previous study, we reported significant differences between brain-activity patterns in regard to correct responses and incorrect responses of a second language at group level (Lei et al., 2018). A reliable quantitative tool for evaluating second-language proficiency based on brain-activation patterns may help people to learn a second language more efficiently. In the present study, we aimed to estimate second-language proficiency using functional brain activity provided by fNIRS data applying machine learning methods. fNIRS data were collected from native Japanese speakers with high/low second-language (English) proficiency and native English speakers with high/low second-language (Japanese) proficiency. Brain activities were recorded by fNIRS when the subjects (speakers) were doing listening-comprehension tasks in English, Japanese, and an unknown language (Chinese). High second-language proficiency and low second-language proficiency is estimated cross participants. To overcome difficulties with estimation across participants and further improve classification performance, the informative features were extracted by using a method of sparse-feature selection. The generalization capability of the machine-learning methods was confirmed by analyzing two independent-validation population groups of native Japanese speakers and native English speakers. In addition, using the label of second-language proficiency classification of the first language and an incomprehensible unknown language was also conducted.

## Materials and Methods

### Participants

All participants in the present study, categorized as native Japanese speakers and native English speakers, were right-handed. The native Japanese speakers were 65 healthy adults (mean age ± SD: 28.5 ± 2.8; range: 24–33; 35 males and 30 females). Based on their TOEIC^®^ Listening & Reading scores, two groups with different English proficiency were recruited. The high-proficiency group contained 32 participants (mean age ± SD: 27.8 ± 2.6; range: 24–32; 18 males and 14 females) with TOEIC^®^ Listening & Reading scores above 700. The low-proficiency group contained 33 participants (mean age ± SD: 29.2 ± 2.8; range: 24–33; 17 males and 16 females) with TOEIC^®^ Listening & Reading scores under 500.

The native English speakers were 66 healthy adults (mean age ± SD: 28.7 ± 2.9; range: 24–33; 34 males and 32 females). They included nationals from Australia, Canada, New Zealand, the United Kingdom, and the United States, who were temporarily staying in Japan for periods ranging from 0.2 to 12 years (mean years ± SD: 3.4 ± 2.6). On the basis of their self-assessments of Japanese proficiency, namely, whether they can speak Japanese or not, the participants were categorized into the high-proficiency group or the low-proficiency group. The high-proficiency group was composed of 31 people (mean age ± SD: 29.3 ± 3.1; range: 24–33; 15 males and 16 females), and low-proficiency group was composed of 35 people (mean age ± SD: 28.1 ± 2.7; range: 24–33; 19 males and 16 females). In addition, all participants did not have experience of learning Chinese.

Data were obtained according to the standards of the internal review board of Research & Development Group, Hitachi, Ltd. Data from volunteers were obtained according to the standards of internal review board on Research & Development Group, Hitachi, Ltd., following receipt of written informed consent.

### Auditory Stimuli and Task Design

Listening comprehension questions from “TOEIC^®^ Listening Test Part 1: Photographs”^1^ were used (ETS, 2005, 2007, 2008, 2012). Each question relates to a photograph with four short explanations. The explanation that most accurately describes the photograph is to be chosen. These listening-comprehension questions were respectively translated into Japanese and Chinese by the native speakers. Sound stimuli were created from the recorded voice of a professional female announcer who is bilingual in Japanese and English, and has learned Chinese as a third language.

**FIGURE 1:**
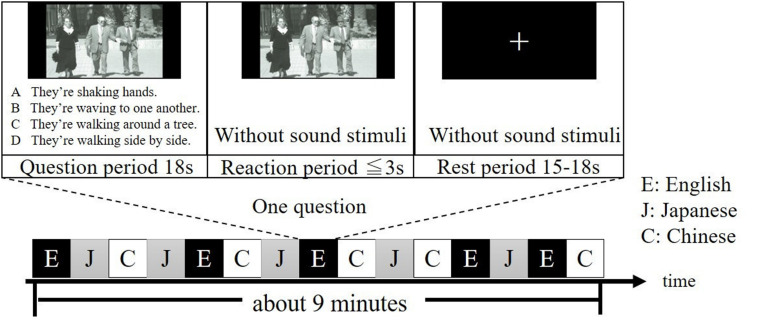
Example of one run of the experiment. Questions in English, Japanese, and Chinese are denoted as E, J, and C, respectively. It takes about 9 min for one run. Questions were selected from the TOEIC^®^ Test New Official Preparation Guide.

All participants were given two runs, each of which contained 15 different questions. During each run, questions in Japanese, English and Chinese were presented five times, respectively, in a pseudo-randomized order (Figure 1). Note that questions in the same language were not given continuously. For each question, a period of 18 s was for presenting the question (question period), a period of less than or equal to 3 s was for answering the question (reaction period) and an arbitrary period between 15 to 18 s was for resting (rest period). The experimental session was conducted in a quiet, dimly lit room. Participants were instructed to look at the photograph on the screen and listen to the four explanations in the question period, answer the question in the reaction period and look at a fixation cross on the screen in the rest period. Specifically, after listening the four explanations of the question, participants were asked to press the button as quick as possible during the reaction period. After pressing the button, the photograph will disappear, and a fixation cross will be shown. Finally, the participants were instructed to silently fix their eyes on the cross and no response was required during the rest period, when they were also asked to think nothing as possible as they can. To ensure that each participant clearly understood the experiment procedure during the on-line tasks, the participants did practice tasks similar to experimental tasks in advance.

### fNIRS Measurement

An optical-topography system (ETG-4000; Hitachi Medical, Japan) was used to measure change in concentration of cerebral cortical hemoglobin. Absorption of near-infrared light at two wavelengths (695 and 830 nm) was measured with a sampling rate of 10 Hz. Two 3 × 5 optode probe sets were placed over the bilateral frontal and temporal areas by referring to the international 10–20 system of electrode placement. Each optode-probe set consists of eight emitters and seven detectors, resulting in 22 measurement channels. The source-detector distance was fixed at 3 cm. For spatial registration, virtual registration (Tsuzuki et al., 2007) was used to register the channel positions in relation to the Montreal Neurological Institute (MNI) standard brain space (Collins et al., 1994; Brett et al., 2002). The anatomical estimation is based on LBPA40 (Shattuck et al., 2008) and Brodmann’s atlas (Rorden and Brett, 2000). The channel positions include regions related to auditory language processing (Friederici et al., 2000; Obrig et al., 2010; Price, 2012; Hall et al., 2013).

### fNIRS Data Preprocessing

For analyzing the fNIRS data, Mathematica (Version 10.0, Wolfram Research, Inc., IL, United States) and Matlab (Version 2017a, Mathworks Inc., Natick, MA, United States) were used. Based on the modified Lambert–Beer law, concentration changes in oxygenated hemoglobin (oxy-Hb) and deoxygenated hemoglobin (deoxy-Hb) on each measurement channel were obtained (Maki et al., 1995). A band-pass filter (0.01–0.8 Hz) was then applied for noise reduction as in a previous study (Sasai et al., 2012; Santosa et al., 2013; Tak and Ye, 2014). The time-continuous data were divided into 33-s language blocks, which consisted of the 18-s question period, the reaction period (less than or equal to 3 s) and the rest period (between 15 and 18 s). After all language blocks were extracted, the baseline was corrected by using linear fitting to a mean signal over 5 s before the task and over the last 5 s of the task. Since optical measurements correspond to the hemodynamic signals, which are an indirect measure of neuronal activity. The hemodynamic signals (representing blood flow) are delayed in relation to the actual neuronal activity (Dehaene-Lambertz et al., 2002). Therefore, in consideration of the delay, the most-informative part of the comprehension during the task period (that is, the amplitude between 5 and 18 s averaged over each extracted language block on each measurement channel) was used to calculate brain activation.

The activation indicator used as an input feature is based on the significant differences between the oxy-Hb and deoxy-Hb signals (Cui et al., 2010). Since fNIRS simultaneously measures the concentration changes of oxy-Hb and deoxy-Hb, this indicator reflects activation strength. For each measurement channel of each participant, the activation indicator is defined as

(1) $$\text{Activation indicator}=\frac{\bar{o\,x\,y}-\bar{d\,e\,o\,x\,y}}{\sqrt{\frac{S_{o\,x\,y^{2}}}{n}+\frac{S_{d\,e\,o\,x\,y^{2}}}{m}}}$$

where $\bar{o\,x\,y}$ and $\bar{d\,e\,o\,x\,y}$ are sample means, *S*_*oxy*_ and *S*_*deoxy*_ are sample standard deviations, and *n* and *m* are sample sizes.

Since there are 44 measurement channels, the number of activation indicators for one participant is 44, and the input feature can be represented as a vector, A = (a_1,…_,a_44_). The number of dimensions of the original input feature is 44.

The label of the participants was re-examined. As a matter of fact, the participants in the high-proficiency group showed a low rate of correct answers, and the participants in the low-proficiency group showed a high rate of correct answers; that is, label proficiency group and label rate of correct answers contradict. To remove ambiguous data, participants whose measurement data did not contradict were further selected from both the native Japanese speakers and native English speakers. After those participants were selected, as for the native Japanese speakers, 20 participants were left in the high-proficiency group, and 20 participants were left in the low-proficiency group; and as for native English speakers, 19 participants were left in the high-proficiency group, and 19 participants were left in the low-proficiency group. The details about number, sex and age are shown in Table 1. As for both the native Japanese speakers and native English speakers, the high- and low-language proficiency groups were age-gender matching groups.

**TABLE 1:** Details of participants after participant selection.

	Native Japanese speakers		Native English speakers	
		
	High-proficiency group	Low-proficiency group	High-proficiency group	Low-proficiency group
*N*	20	20	19	19
Female/male	8/12	10/10	6/13	8/11
Age (mean ± SD)	28.1 ± 2.6	29.4 ± 2.9	29.2 ± 2.9	28.5 ± 2.4

### Algorithm Evaluation

The following conventional methods, which were shown to be promising by various classification studies, were used to classify the language proficiency into the high or low group.

• Support Vector Machine (SVM)
• Sparse Logistic Regression (Yamashita, 2009) (SLR)
• K-Nearest-Neighbors based on Euclidean distance of original input features (KNN, *K* = 5).

Using brain-activation vectors [for example, A = (a_1,…_,a_44_)] for classifying each participant into the high or low groups was evaluated. Concretely, a support-vector machine (SVM) is considered to be a promising and popular algorithm among those used in classification studies, and it has been used in a variety of fNIRS studies (Li et al., 2016; Hosseini et al., 2018). Moreover, a SVM has already been used to examine the diagnostic potential of neuroimaging for a range of psychiatric disorders (Nieuwenhuis et al., 2012; Orrù et al., 2012). A SVM with a linear kernel was adopted for the binary-classification problem. The algorithm known as sparse logistic regression (SLR) (Yamashita, 2009) is an extension of logistic regression to automatically select features related to a label. Logistic regression is extended to a Bayesian framework by using a technique known as automatic relevance determination (ARD) from the neural-network literature. By combining logistic regression with ARD, SLR is obtained. SLR is effective for removing irrelevant features, such that their associated weights are automatically set to zero, leading to a sparse weight vector for classification. In the implementation of this study, default values in the SLR toolbox were used to do the classification. K-nearest neighbor(s) (KNN) using majority voting (Duda et al., 2012) was used for classification. In this study, K was fixed to 5. K-nearest neighbors defines the label of test data by looking at the K-closest training data in the feature space. And it is sensitive to the local structure of the data.

Leave-one-out cross validation (LOOCV) was applied for cross validation. In detail, the data are divided into N folds (*N* = 40 for the native Japanese speakers; *N* = 38 for the native English speakers). In each leave-one-out cross-validation fold, all except one participant (N-1) were used as training data; the one participant left out was used as test data to determine which group the participant came from. This process is repeated once for each participant.

The classification accuracy of second language proficiency was computed to verify the estimation performance of the algorithm. A confusion matrix contains information about actual and predicted classifications done by a classification system. Performance of such a system is commonly evaluated using the data in the matrix.

(2) $$\text{Accuracy}=\frac{T\,P+T\,N}{T\,P+T\,N+F\,P+F\,N},$$

where *TP* is the number of true positives, *TN* is the number of true negatives, *FP* is the number of false positives, and *FN* is the number of false negatives.

### Informative-Feature Selection

Given the small number of data sets and the high dimensions of the data, to further improve classification accuracy, feature selection or feature extraction is necessary (Guyon and Elisseeff, 2003; Akama et al., 2014). By selecting informative features, the machine-learning algorithm can give stable results and the physical interpretations of selected features are also important and worth discussing by means of neuroscience.

In this study, sparse canonical correlation analysis (SCCA) is applied to select the informative features. SCCA identifies sparse linear combinations of two sets of highly correlated variables (Witten et al., 2009). It has been shown to be useful in the analysis of high-dimensional neuroimaging data, namely, when two sets of variables are available for the same set of samples (Yahata et al., 2016).

Specifically, *N* observations (participants) of paired variables X∈R^*d1*^ and Y∈R^*d2*^ are given, *X* is an N × d1 matrix comprising the first set of variables, and *Y* is an N × d2 matrix comprising the second set of variables. L1-norm SCCA can be formulated as

(3) $$\max_{v_{X},v_{Y}}v_{X}^{T}\,X^{T}\,Y\,v_{Y}\,\text{subject to}\,{||v_{X}||}_{1}^{2}\leq \lambda_{X},{||v_{Y}||}_{1}^{2}\leq \lambda_{Y},{||v_{X}||}_{2}^{2}\leq 1,{||v_{Y}||}_{2}^{2}\leq 1,$$

where hyperparameters λ_*X*_ and λ_*Y*_ indicate the sparseness of projection vectors *v*_*X*_ and *v*_*Y*_, respectively. The projection matrices are *v*_*X*_ ∈ *R*^*d*1×*m*^ and *v*_*Y*_ ∈ *R*^*d*2×*m*^, where m = min (d1, d2). In this study, *X* is the input feature, and *Y* is the label for language proficiency. Before input to SCCA, the training set is centered to have zero mean and scaled to have unit variance.

To select the common informative feature, the data sets for the second language were applied, and the LOOCV described above was used for cross validation. A feature selected from more than 95% of participants during the leave-one-out procedure was defined as a common informative feature. That is, a feature was selected when it was shown more than 38 times by the Japanese speakers (*N* = 40) and more than 36 times by the English speakers (*N* = 38). After the common informative features were selected, machine-learning methods were used to classify language proficiency to confirm that classification accuracy has been improved.

## Results

### Classification Performance

**FIGURE 2:**
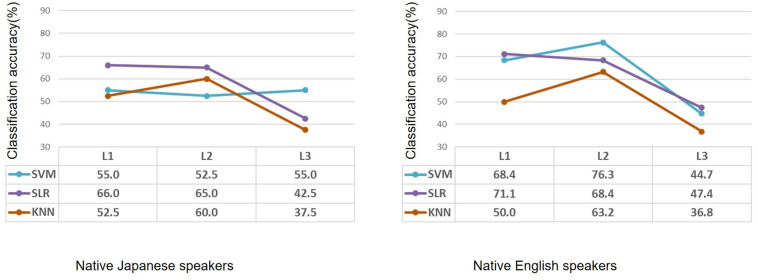
Performance of classification by using machine learning methods of participants with high or low second-language proficiency in the case of native Japanese speakers and native English speakers. Based on the same second-language-proficiency label, classification results of the first language and third (unknown) language are also shown. L1: first language; L2: second language; L3: third (unknown) language.

Classification performance for the native Japanese speakers and the native English speakers is shown in Figure 2. Classification accuracies of the SVM for the native Japanese speakers were 55.0%, 52.5%, and 55.0% in terms of first language (L1), second language (L2) and third (unknown) language (L3), respectively, which show low classification accuracy. When SLR was used, classification accuracies were 66.0%, 65.0%, and 42.5% for the three languages, respectively; namely, classification of L1 and L2 showed higher accuracy than that for L3. When KNN was used, classification accuracies were 52.5%, 60.0%, and 37.5%; that is, only classification of L2 showed higher accuracy. For the native English speakers, classification accuracies of SVM were 68.4%, 76.3%, and 44.7% for L1, L2, and L3, respectively; that is, classification of L2 showed higher accuracy. When SLR was used, classification accuracies were 71.1%, 68.4%, and 47.4% for the three languages, respectively. When KNN was used, classification accuracies were 50.0%, 63.2%, and 36.8%, respectively. SLR showed the highest classification accuracy for the second language in the case of the Japanese speakers, and SVM showed the highest classification accuracy for the second language in the case of the English speakers.

The SVM showed unstable classification results: it cannot classify language proficiency in the case of the native Japanese speakers. SLR showed unexpected high classification accuracy when classifying L1. Using the label of second-language proficiency, SLR showed the highest classification accuracy for L1 in the case of both the native Japanese speakers and native English speakers. K-nearest neighbor showed reasonable classification results; namely, classification accuracy for L2 is higher, and that for L1 and L3 is near to chance level.

### Informative Features Shared Between High- and Low-Language-Proficiency Groups

**FIGURE 3:**
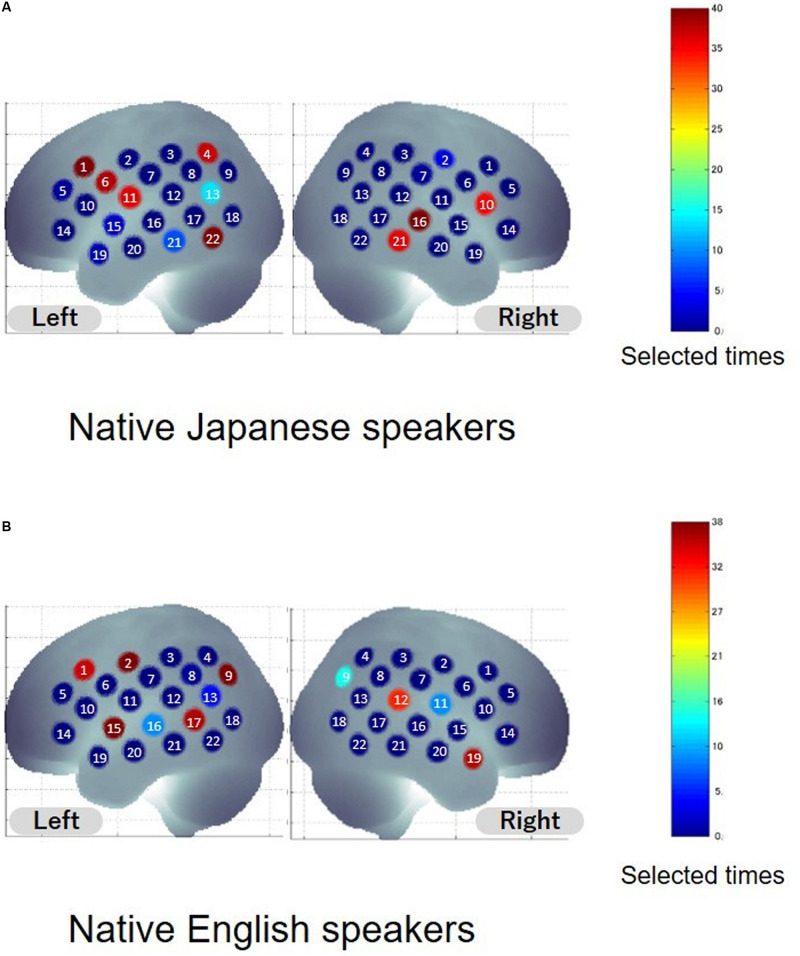
Distribution of selected features. Colors indicate the number of times the channel was selected as the feature. The number on the channel indicates the channel number. **(A,B)** Show the results for native Japanese speakers and native English speakers, respectively.

To extract informative features that can improve classification accuracy, sparse canonical correlation analysis was used. The same leave-one-out cross validation (LOOCV) procedure was adopted to select the features. Common informative features were defined as features selected from more than 95% of the participants. Spatial distribution of informative features is shown in Figure 3, and anatomical information about the features is listed in Table 2. As for the native Japanese speakers, the selected common informative features correspond to channel 1, channel 6, and channel 22 on the left hemisphere and channel 16 on the right hemisphere. The anatomical information about these features indicate the pars opercularis, part of Broca’s area, left precentral gyrus, left inferior temporal gyrus and right superior temporal gyrus. As for the native English speakers, the selected common informative features correspond to channel 2, channel 9, channel 15, and channel 17 on the left hemisphere and channel 19 on the right hemisphere. The anatomical information about these features indicate the left postcentral gyrus, left angular gyrus, part of Wernickes’s area, left superior and middle temporal gyrus and right middle temporal gyrus.

**TABLE 2:** Spatial and anatomical information of selected features.

		MNI	
		coordinates	Anatomical information
	Channels	x,y,z	
Japanese	Left Ch 1	−50, 22, 38	Left middle frontal gyrus (BA 44)
speakers	Ch 6	−61, 7, 28	Left precentral gyrus (BA 6)
	Ch 22	−61, −62, −8	Left inferior temporal gyrus (BA 37)
	Right Ch 16	71, −25, 4	Right superior temporal gyrus (BA 21)
English	Left Ch 2	−60, –7, 43	Left postcentral gyrus (BA 4)
speakers	Ch 9	−52, −72, 35	Left angular gyrus (BA 39)
	Ch 15	−62, 2, 1	Left superior temporal gyrus (BA 48)
	Ch 17	−67, −50, 6	Left middle temporal gyrus (BA 21)
	Right Ch 19	58, 10, −18	Right middle temporal gyrus (BA 21)

**FIGURE 4:**
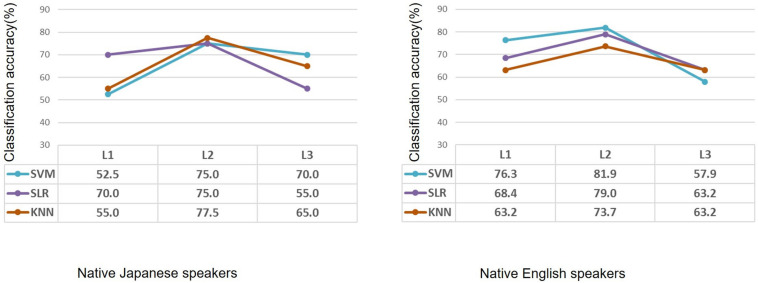
Classification accuracy after feature selection in the case of native Japanese speakers and native English speakers using machine learning methods. Based on the same second-language-proficiency label, classification results of the first language and third (unknown) language are also shown. L1: first language; L2: second language; L3: third (unknown) language.

After feature selection, the informative features were used to classify each participant into the high-proficiency group or the low-proficiency group. Classification accuracy for L1, L2, and L3 in the case of the native Japanese speakers and the native English speakers is shown in Figure 4. As for the native Japanese speakers, when SVM was used, classification accuracy for L2 was the highest, i.e., 75%. When SLR was used, classification accuracies were 70.0%, 75.0%, and 55.0% for L1, L2, and L3, respectively; similarly, the accuracy was highest for L2. When KNN was used, classification accuracies were 55.0%, 77.5%, and 65.0%. As for the native English speakers, when SVM was used, classification accuracies for L1, L2, and L3 were 76.3%, 81.9%, and 57.9%, respectively. When SLR was used, classification accuracies were 68.4%, 79.0%, and 63.2% for L1, L2, and L3, respectively. When KNN was used, classification accuracies were 63.2%, 73.7%, and 63.2%; namely, classification accuracy was highest for L2.

After feature selection, as expected, classification accuracy for the second language was improved; meanwhile, classification accuracies for the first and unknown languages tend to be at the chance level. On the other hand, the SVM showed higher accuracy for L3 in the case of the native Japanese speakers and for L1 in the case of the native English speakers. SLR also showed higher accuracies for L1 in both cases. When K-nearest neighbor was used, classification accuracy tended to be reasonable; that is, it showed higher classification accuracy for L2. After feature selection, all the algorithms showed higher classification accuracy for L2. These results suggest that the informative features, which are related to second-language proficiency, are important for improving classification accuracy.

## Discussion

In this study, machine-learning methods—using activation patterns in fNIRS data— were used to classify individuals with high second-language proficiency or low second-language proficiency, in the case of both native Japanese speakers and native English speakers. After feature selection, all methods showed higher classification accuracy for the second language, suggesting that the validity of feature selection. Also, the activation patterns of frontal-temporal region are important indicators to estimate individual language proficiency.

In the field of neuroimaging studies applying machine learning, it is believed that to achieve better classification accuracy, informative features must be extracted (Norman et al., 2006; De Martino et al., 2008; Pereira et al., 2009; An et al., 2017). The higher classification performance demonstrated in this study indicates the validity of feature selection. Optimal feature extraction avoids over-fitting and eliminates the effects of noisy variables that are irrelevant to the classification problem. How to extract intrinsic features is an important research focus. SCCA was used as one of the methods for extracting informative features for individual estimation (Yahata et al., 2016). CCA can derive projection vectors that have maximum correlation with desired labels (e.g., a label for language proficiency). Using L1-norm regularization will lead to sparse solutions. As a result, features only related to desired labels can be extracted, so label-unrelated variables can be eliminated. Conventional methods of feature selection need careful engineering and considerable domain expertise to design a feature extractor that transforms raw data into an appropriate feature vector. SCCA allows an input to be composited from raw data; thus, it makes it possible to automatically extract the informative features required for the classification task.

Analyzing the most-discriminative features shared between high- and low-language-proficiency groups revealed that native Japanese speakers and native English speakers utilize different specific brain regions, but they show the same tendency, that is, Broca’ s area, Wernicke’ s area and the temporal cortex. The reason for activation of different specific brain regions may be due to the differences between brain shapes of native Japanese speakers and native English speakers; consequently, specific brain regions may deviate during spatial registration of measurement channels (Chee et al., 2011). Previous studies have found evidence that the two languages extensively overlap in regard to the classical language areas, namely Broca’s area and Wernicke’s area. Specifically, a variety of regions, including the left frontal region (Price et al., 1999; Lehtonen et al., 2005; Abutalebi and Green, 2008) and the bilateral supramarginal gyri (Price et al., 1999) have been observed to be involved in bilingual language comprehension and processing. Those studies also suggested that no single region is responsible for language comprehension and processing. Moreover, multiple studies have suggested that the bilateral temporal-frontal network is involved in processing during auditory language comprehension (Friederici, 2002; Price, 2012; Fengler et al., 2016). Concretely, syntactic and semantic information are processed predominately by the left hemisphere, while processing of prosodic information occurs predominately in the right hemisphere (Friederici et al., 2000; Friederici, 2002). Studies on sentence-comprehension tasks also reported left laterality plays a primary and significant role in language comprehension (Harrington et al., 2006; Sanjuán et al., 2010; Niskanen et al., 2012). In the present study, the brain region of selected features are consistent with the previous findings; namely, multiple cortical regions in a temporal-frontal network were observed to be related to language comprehension irrespective of native language, and the informative features in these brain regions play an important role in improving classification accuracy.

Using the label of second-language proficiency, classification of L1 and L3 results in a higher classification accuracy than the chance level. One possible explanation of this result is the relation between the second-language ability and the native-language ability (Brevik et al., 2016; Guo, 2018). In addition, during the fNIRS-measurement experiment, the psychological stressors of the high-language-proficiency group and the low-language-proficiency group may differ. Since all the languages were randomly presented, the tests for L1 and L3 may be affected by the different psychological stressors.

## Conclusion

Machine-learning methods were used for distinguishing second-language proficiency individually for both native Japanese speakers and native English speakers. By extracting informative features, the machine-learning methods showed higher classification accuracy for the second language. The informative features showed the effectiveness of feature selection in improving classification accuracy. Moreover, brain-activation patterns measured by fNIRS have the potential to serve as biomarkers for identifying language proficiency. Finally, the same approach could potentially be used with other biological data with similar characteristic to those of fNIRS data.

## Data Availability Statement

The datasets generated for this study are available on request to the corresponding author.

## Ethics Statement

The studies involving human participants were reviewed and approved by internal review board on Research & Development Group, Hitachi, Ltd. The patients/participants provided their written informed consent to participate in this study.

## Author Contributions

HS, ID, and TM conceived and designed the study. HS, TM, ID, and ML conducted the experiment. ML, TM, and HS carried out the analysis of the data. ML wrote the manuscript. All the authors reviewed the manuscript.

## Conflict of Interest

ML and TM was employed by the company Hitachi, Ltd. The remaining authors declare that the research was conducted in the absence of any commercial or financial relationships that could be construed as a potential conflict of interest.

## Footnotes

1 Written pledge was submitted to copyright owner IIBC (The Institute for International Business Communication) and permission was obtained from the copyright owner. Questions were selected from the TOEIC^®^ Test New Official Preparation Guide vol. 1 (2005), vol. 2 (2007), vol. 3 (2008), and vol. 5 (2012) published by IIBC, and copyrighted by Educational Testing Service (ETS). TOEIC is a registered trademark of ETS. This publication is not endorsed or approved by ETS.
